# Improved Growth Velocity Using a New Liquid Human Milk Fortifier in Very Low Birth Weight Infants: A Multicenter, Retrospective Study

**DOI:** 10.1055/a-2527-4638

**Published:** 2025-03-06

**Authors:** Fernando Moya, Jennifer Fowler, Adrian Florens, Jennifer Dombrowski, Olivia Davis, Tiffony Blanks, Austin Gratton

**Affiliations:** 1 Betty Cameron Children's Hospital and University of North Carolina Medical School, Wilmington, North Carolina; 2 East Carolina University Health Medical Center, Greenville, North Carolina; 3 Department of Neonatology, Kidz Medical Services, Palm Beach Children's Hospital, Palm Beach, Florida; 4 Novant Health New Hanover Regional Medical Center, Wilmington, North Carolina

**Keywords:** human milk fortification, human milk, growth velocity, postnatal nutrition, very low birth weight infants

## Abstract

**Objective:**

This study aimed to compare growth outcomes and tolerance among very low birth weight (VLBW) infants receiving a new, liquid human milk fortifier (LHMF-NEW) or a human milk fortifier-acidified liquid (HMF-AL).

**Study Design:**

Retrospective, multicenter study of 515 VLBW infants in three regional neonatal intensive care units. The primary objective was to compare growth velocity (g/kg/d) during fortification between groups by repeated measures regression. Secondary outcomes of interest were feeding tolerance and the incidence of late-onset sepsis, necrotizing enterocolitis, and metabolic acidosis. Student's *t* , analysis of variance, Wilcoxon, and Kruskal–Wallis tests were used for numeric variables, or chi-squared and Fisher's exact test for categorical variables.

**Results:**

No demographic differences were identified between the groups (HMF-AL, *n* = 242; LHMF-NEW, *n* = 273). Growth velocity during fortification was significantly higher in the group receiving LHMF-NEW, despite relatively similar total fluid, calorie, or protein intake ( *p* = 0.001). Feeding intolerance was comparable between fortifiers. Necrotizing enterocolitis and late-onset sepsis did not differ between groups and metabolic acidosis was diagnosed less frequently with the LHMF-NEW. Anthropometric measures at discharge and length of stay were comparable.

**Conclusion:**

Infants receiving human milk fortified with the LHMF-NEW had faster growth velocity during fortification, similar tolerance, and less metabolic acidosis compared with an earlier cohort of infants who received human milk fortified with an HMF-AL.

**Key Points:**

- Among VLBW infants, using an LHMF-NEW resulted in a faster growth velocity in weight during several weeks of fortification than using the previous HMF-AL.
- The incidence of feeding intolerance (stopping feeds >8 hour) in any given week of fortification was low and not different between groups. Also, late-onset sepsis and necrotizing enterocolitis were uncommon with no differences between groups, whereas the incidence of metabolic acidosis was lower in infants receiving the LHMF-NEW.
- No differences in length of stay or anthropometrics at discharge were identified.

Human milk is the preferred source of nutrition to feed all infants. 1 Most preterm infants receive their mother's own expressed milk (MOM) or donor human milk (DHM) as their main source of feeding. 2 The protein content of human milk as well as that of other nutrients may not be sufficient to meet the higher nutritional requirements of preterm infants, especially infants of very low birth weight (VLBW). 3 The American Academy of Pediatrics and other organizations, therefore, recommend adding specifically designed human milk fortifiers to either MOM or DHM to promote better growth. 4 5 Implementing best feeding practices for VLBW infants may help with reduce short- and long-term consequences of poor growth. Human milk fortifiers can be of human milk or bovine milk origin and have been in use for several decades. These fortifiers have been studied extensively, and there is insufficient evidence to support the choice of human milk-based fortifiers over bovine milk-derived products. 6 7 8 9 10 Liquid bovine milk-derived fortifiers have been available for over a decade and their composition has been adjusted to increase their protein content and add long-chain polyunsaturated fatty acids. 11 12 A better understanding of many of the macro- and micronutrients found in human milk has also resulted in other modifications of these fortifiers to improve their ability to support nutrition and growth of preterm infants. 3 13

Consequently, it is important to continue evaluating clinical outcomes, particularly growth, among populations of infants receiving these fortifiers. Recently, a new, liquid human milk fortifier became available (LHMF-NEW, Enfamil Liquid Human Milk Fortifier High Protein, Enfamil Liquid Human Milk Fortifier Standard Protein, Mead Johnson Nutrition, Evansville, IN). This new fortifier comes in two preparations with different protein content, namely High Protein or Standard Protein ( Table 1 ). An important change is the use of a manufacturing process to maintain sterility that does not lower the pH of the commercial product. To date, there are no reports of clinical experiences with the new fortifier compared with a population of infants who received the previous acidified liquid human milk fortifier from the same manufacturer (HMF-AL). Therefore, we decided to compare growth outcomes, feeding tolerance, and the incidence of other morbidities, including metabolic acidosis, in a population of VLBW infants receiving these fortifiers.

**Table 1:** Macro and micronutrient composition of the fortifiers under study a

	HMF-AL	LHMF-NEW High protein and (standard)
Total calories	100	100
Protein, g	4	4 (3.4)
Carbohydrate, g	8.1	7.9 (8.7)
Fat, g	6	6
DHA, mg	24	24
ARA, mg	38	38
Calcium, mg	145	145
Phosphorus, mg	80	80
Sodium, mg	57	57
Iron, mg	1.91	1.9
Zinc, mg	1.37	1.37
Vitamin A, IU	1250	1240
Vitamin D, IU	210	210
Vitamin E, IU	6.2	6.2
Potassium, mg	98	98
Magnesium, mg	5.3	5.3
Osmolality, mOsm/kg H _2_ O	336	350 (330)
pH	4.7	6

Abbreviations: ARA, arachidonic acid; DHA, docosahexaenoic acid; HMF-AL, human milk fortifier- acidified Liquid; LHMF-NEW, new, liquid human milk fortifier.

a Per 100 Kcal of fortified preterm human milk fortified to 24 Kcal/ounce. Values in parenthesis reflect those in the standard fortifier preparation.

## Materials and Methods

### Study Design and Population

We conducted a retrospective study in three neonatal intensive care units (NICU's), two in North Carolina (Betty Cameron Children's Hospital, BCCH; East Carolina Health, ECH) and one in Florida (Palm Beach Children's Hospital, PBCH). The institutional review boards at each institution approved the study and exempted it from acquiring informed consent given its retrospective nature and use of deidentified data. The entry criteria for the study were as follows: (1) gestational age ≤ 32 weeks, (2) birth weight ≤ 1,500 g, (3) receiving mother's own or donor milk (no formula), (4) enteral feedings ≥ 80 mL/kg/d. Exclusion criteria were: (1) intake of any formula or fortified breast milk using a bovine-derived fortifier prior to study entry, (2) diagnosis of a significant chromosomal abnormality or a condition incompatible with progressive enteral feeding, (3) significant depression after delivery (Apgar score < 4 at 5 minutes), (4) use of pharmacologic doses of corticosteroids within 3 days prior to starting fortification (because of their potential role in stunting growth), (5) undergoing fluid restriction (fluid intake < 120 mL/kg/d) at start of fortification, (6) use of probiotics. These entry and exclusion criteria were chosen since they were used in previous controlled trials of fortifiers that recruited a relatively similar population. 11 12 14 Moreover, by choosing a population of VLBW infants, there would be a period of fortification long enough to potentially identify differences in clinical outcomes between the fortifiers under study.

### Human Milk Fortifiers and Neonatal Intensive Care Units Feeding Practices

All three NICU's initiated the use of the LHMF-NEW during 2020 or 2021 (BCCH, September 2020; ECH, January 2021; PBCH, August 2020) and had previously utilized the HMF-AL of the same manufacturer. Fortifiers were used per manufacturer's recommendations. One center's feeding protocol (PBCH) used a human milk-based product (HMBP, 20 Kcal/oz, Prolacta Bioscience, Duarte, CA) for infants below 32 weeks, if MOM was unavailable. In this center, fortification was done at first with HMBP and progressed to the bovine fortifier past 30 to 32 weeks of corrected gestational age.

All three centers used their own feeding guidelines ( Supplementary Material S1 , available in the online version). In brief, all of them began with a variable period of “trophic feeds” between 10 and 20 mL/kg/d of either MOM or DHM. In one center (PBCH), HMBP (20 cal/oz) was used to begin “trophic feeds.” These were subsequently advanced after a variable number of days by approximately 20 mL/kg/d and fortification was started when an enteral intake above 70 to 100 mL/kg/d was reached. If HMBP had been supplied, this was switched to either HMF-AL or LHMF-NEW after approximately 7 days of use or at 32 weeks' corrected gestational age. Administration of parenteral nutrition and lipid emulsions was decreased and eventually stopped in all three centers when infants reached an enteral intake of approximately 90 to 120 mL/kg/d.

### Data Collection and Study Outcomes

Data were abstracted from medical records into deidentified case report forms. Total fluid intake, concomitant medications, and laboratory values were recorded. Total calories and protein intake were estimated for each week while infants were receiving fortification by adding the actual intake in parenteral nutrition (if still receiving it) plus the estimated intake in MOM or DHM, including any intake added by the fortifiers. Whereas the exact proportion of MOM versus DHM used daily was not consistently documented, whenever DHM was listed as being fed, an estimated protein intake of 0.9 g/dL was used if DHM was fed or 1.1 g/dL if MOM was fed. When both were used, estimations assumed that they were mixed in approximately equal proportions.

Growth measurements were done according to NICU policies and were recorded weekly while receiving fortification. Length was measured with either tapes or length boards according to NICU practices. The Z-scores for weight, length, and head circumference were determined using the Fenton growth curves. 15 Growth velocity was calculated using the exponential method as previously described. 16 Feeding intolerance was defined as stopping feeding for more than 8 hours on any given day. 11 This was assessed and reported by week of fortification and any one infant could have experienced feeding intolerance in different weeks of fortification. Metabolic acidosis was diagnosed if there was a serum bicarbonate < 18 mEq/L or a base deficit below −6 in an electrolyte or blood gas determination obtained at any time after starting the fortification study period. 11 14 Also, whether this was addressed by stopping/modifying fortification or with the use of a therapeutic agent was consigned. Late-onset sepsis was diagnosed if the infant had positive blood cultures that were not deemed a contaminant and the infant received antibiotic treatment for more than 2 days. Necrotizing enterocolitis (NEC) was recorded when infants had a confirmed stage II or higher using Bell's criteria. 17 We only recorded those episodes of late-onset sepsis or NEC diagnosed after fortification had been initiated. Whether metabolic acidosis occurred within 48 hours of the diagnosis of sepsis was also determined. Whereas the diagnosis of bronchopulmonary dysplasia was not recorded due to the potential variability in its definition in the NICU's participating in this study, we collected information on whether participants were receiving supplemental oxygen, noninvasive respiratory support (nasal cannula or continuous positive airway pressure [CPAP]), or mechanical ventilation during the fortification period.

### Statistical Analysis

We used a convenience sample of infants who received the LHMF-NEW during 12 to 15 months after its initiation at each center. For comparison, we collected data from infants who received the HMF-AL for a similar period of time before switching to the new preparation. We allowed a period of 1 to 3 months between the change from the HMF-AL to the LHMF-NEW to avoid collecting data from infants who may have received both. The proportion of all VLBW infants admitted to their respective NICU's during the study period that were entered into this study were 70% for BCCH, 64% for ECH, and 34% for PBCH.

Clinical characteristics are reported as median and 25 ^th^ to 75 ^th^ interquartile ranges. Other data are reported as indicated. Comparison between groups was performed using Student's *t* , analysis of variance, Wilcoxon, and Kruskal–Wallis tests where applicable for numeric variables; chi-squared and Fisher's exact test were used where appropriate for categorical variables. A repeated measures regression (using lme4 version 1.1–35.5, R version 4.4.1) was used to compare growth velocity between groups only including infants who received either fortifier throughout the first 4 weeks of fortification ( *N* = 308). Gestational age, fortifier group, location, sex, and use of supplemental oxygen or respiratory support (see results) were incorporated into the model.

## Results

### Clinical Characteristics

A total of 515 infants were included ( Tables 2 and 3 ). Infants in the study were on average approximately 28 to 29 weeks of gestational age (HMF-AL= 28.6 [26.4, 30.2] and LHMF-NEW = 28.5 [27.0, 30.0], median [25–75 IQR (interquartile range)]) and approximately 1,100 g birth weight (HMF-AL = 1,100 [870, 1,280] and LHMF-NEW = 1,070 [870, 1,270], median [25–75 IQR]). A small proportion in either group were growth-restricted at birth (HMF-AL 10.7%, LHMF-NEW 12.8%). In both groups, enteral feeds began at approximately 3 days after birth (HMF-AL = 3.0 [1.0, 6.0] and LHMF-NEW = 3.0 [1.0, 6.0], median [25–75 IQR]), infants received approximately 6 days of parenteral nutrition before fortification (HMF-AL = 6.0 [5.0, 9.0] and LHMF-NEW= 6.0 [5.0, 9.0], median [25–75 IQR]), and a bovine milk-derived fortifier was generally started approximately 7 days after birth (HMF-AL = 7.0 [6.0, 12.0] and LHMF-NEW = 7.0 [6.0, 12.0], median [25–75 IQR], although in one center (ECH) feedings and fortification were started much sooner ( Table 2 ). DHM was used in about 2/3 of the infants in either group. In one NICU (PBCH) over half of all infants received HMBP (HMF-AL, 53%; LHMF-NEW, 56%) before switching to bovine-derived fortifiers as described in Methods.

**Table 2:** Clinical characteristics of infants from each center a

	Betty Cameron	Palm Beach	East Carolina	*p*
*N*	179	92	244	
Gestational age, wk	28.5 [26.4, 30.2]	29.0 [27.5, 30.0]	28.3 [26.4, 30.0]	0.056
Female sex, *N* (%)	86 (48.0)	44 (47.8)	141 (57.8)	0.076
Birth weight, g	1,108 [910, 1,290]	1,152 [970, 1,320]	1,004 [814, 1,253]	0.002
Birth length, cm	37.0 [33.0, 38.5]	37.0 [35.0, 39.0]	36.0 [32.5, 38.0]	0.01
Birth FOC, cm	25.7 [24.0, 27.3]	26.2 [25.0, 27.1]	25.0 [23.0, 26.5]	<0.001
SGA, *N* (%)	20 (11.2)	10 (10.9)	31 (12.7)	0.82
Multiple births, *N* (%)	36 (20.1)	30 (32.6)	49 (20.1)	0.033
Apgar at 5 min	8.0 [7.0, 9.0]	8.0 [8.0, 9.0]	7.0 [6.0, 8.0]	<0.001
C-section, *N* (%)	136 (76.0)	74 (80.4)	196 (80.3)	0.51
Donor milk used (%)	120 (67.0)	61 (66.3)	147 (60.2)	0.30
Day when birth weight regained	8.0 [6.0, 10.0]	8.0 [6.0, 10.5]	9.0 [7.0, 13.0]	0.002
Day when nutritive enteral feeds started b	7.0 [5.0, 9.0]	4.0 [3.0, 5.0]	4.0 [3.0, 4.0]	<0.001
Days of PN before study day 0	8.5 [7.0, 12.7]	9.0 [7.0, 11.0]	5.0 [5.0, 6.0]	<0.001
Days of age at start of bovine fortification	10.0 [7.0, 14.0]	14.0 [10.7, 23.0]	6.0 [5.0, 7.0]	<0.001
In oxygen or respiratory support c at 4 wk of fortification, *N* (%)	54 (30.2%)	33 (35.9)	109 (44.7)	0.009
Sepsis, *N* (%)	5 (2.8)	4 (4.3)	31 (12.7)	<0.001
Necrotizing enterocolitis, *N* (%)	7 (3.9)	2 (2.2)	12 (4.9)	0.61

Abbreviations: C-section, cesarean section; FOC, fronto occipital circumference; IQR, interquartile range; PN, parenteral nutrition; SGA, small for gestational age.

a All values presented as median [27–75 IQR], unless otherwise indicated.

b Enteral feeds after “trophic feeds.”

c Nasal cannula, continuous positive airway pressure, or mechanical ventilation.

**Table 3:** Clinical characteristics of infants receiving either fortifier a

	HMF-AL	LHMF-NEW	*p*
*N*	242	273	
Gestational age	28.6 [26.4, 30.2]	28.5 [27.0, 30.0]	0.91
Female sex, *N* (%)	116 (47.9)	154 (56.4)	0.06
Birth weight, g	1,100 [870, 1,280]	1,070 [870, 1,270]	0.65
Birth length, cm	36.0 [33.0, 38.5]	36.5 [33.5, 38.5]	0.54
Birth FOC, cm	25.5 [23.5, 27.0]	25.5 [24.0, 27.0]	0.79
SGA, *N* (%)	26 (10.7)	35 (12.8)	0.58
Multiple births, *N* (%)	60 (24.8)	55 (20.1)	0.24
Apgar at 5 min	8.0 [7.0, 8.0]	8.0 [7.0, 8.0]	0.30
C-section, *N* (%)	185 (76.4)	221 (81.0)	0.25
Donor milk used, *N* (%)	161 (66.5)	167 (61.2)	0.24
Day nutritive enteral feeds started b	3.0 [1.0, 6.0]	3.0 [1.0, 6.0]	0.96
Days of PN before study day 0	6.0 [5.0, 9.0]	6.0 [5.0, 9.0]	0.32
Days of age at fortification	7.0 [6.0, 12.0]	7.0 [6.0, 12.0]	0.58
In oxygen or respiratory support c at 4 wk of fortification, *N* (%)	79 (32.6)	117 (42.9)	0.02
Sepsis, *N* (%)	23 (9.5)	17 (6.2)	0.22
Necrotizing enterocolitis, *N* (%)	10 (4.1)	11 (4.0)	1.0

Abbreviations: C-section, cesarean section; FOC, fronto occipital circumference; IQR, interquartile range; HMF-AL, human milk fortifier-acidified liquid; LHMF-NEW, new, liquid human milk fortifier; SGA, small for gestational age.

a Data are expressed as (median [25–75 IQR]) or % as indicated.

b Enteral feeds after “trophic feeds.”

c Nasal cannula, continuous positive airway pressure, or mechanical ventilation.

Several significant differences in clinical characteristics were detected between NICUs ( Table 2 ); however, when infants were grouped by the HMF received (HMF-AL vs LHMF-NEW), no significant differences were detected with the exception of more frequent use of supplemental oxygen/respiratory support in the LHMF-NEW group at 4 weeks of fortification (HMF-AL 32.6% vs. LHMF-NEW 42.9%, *p* = 0.02, Table 3 ). Even though culture-proven sepsis differed among centers ( Table 2 ), it was diagnosed in 9.5 and 6.2% of infants in the HMF-AL and LHMF-NEW groups, respectively ( *p* = 0.22, Table 3 ). Confirmed NEC was uncommon in both groups (HMF-AL 4.1%, LHMF-NEW 4.0%). The diagnosis of metabolic acidosis decreased in all centers although the frequency of this condition was different among NICUs (BCCH: HMF-AL 17% vs. LHMF-NEW 6.6%, *p* = 0.05; PBCH: HMF-AL 21% vs. LHMF-NEW 2%, *p* = 0.005; ECH: HMF-AL 73.1% vs. LHMF-NEW 60.6%, *p* = 0.05). Although all values favored the LHMF-NEW group, there were no significant differences in median [25–75% IQR] length of stay (HMF-AL 68 [48, 91] days vs. LHMF-NEW 66 [48, 54] days) or corrected gestational age at discharge (HMF-AL 38.5 [37.0, 40.6] weeks vs. LHMF-NEW 38.1 [36.4, 40.5] weeks).

### Intake and Feeding Tolerance

**Fig. 1:**
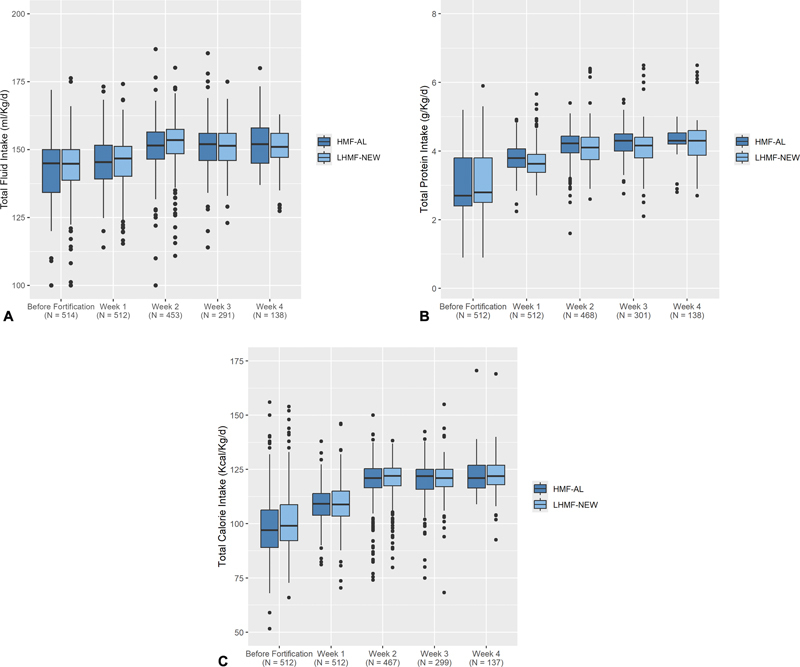
( **A** ) Total fluid intake over the first 4 weeks of fortification for the groups receiving HMF-AL (blue) or LHMF-NEW (light blue) shown as median and 25 to 75 IQR, vertical lines represent the range. The total *N* per week of fortification is shown in the *x* -axis. There were no significant differences. ( **B** ) Total protein intake over the first 4 weeks of fortification for the groups receiving HMF-AL (blue) or LHMF-NEW (light blue) is shown as median and 25 to 75 IQR, vertical lines represent the range. The total *N* per week of fortification is shown in the *x* -axis. Significant differences in HMF-AL versus LHMF-NEW (median [IQR]): Week 1, 3.80 [3.53, 4.08] versus 3.63 [3.38, 3.90], *p* < 0.001; Week 2, 4.22 [3.95, 4.43] versus 4.10 [3.75, 4.40], *p* = 0.015; Week 3, 4.30 [4.00, 4.50] versus 4.16 [3.80, 4.40], *p* = 0.006. ( **C** ) Total calorie intake over the first 4 weeks of fortification for the groups receiving HMF-AL (blue) or LHMF-NEW (light blue), shown as median and 25 to 75 IQR, vertical lines represent the range. The total *N* per week of fortification is shown in the *x* -axis. Significant differences in HMF-AL versus LHMF-NEW (median [IQR]): before fortification, 97.0 [89.00, 106.2] versus 99.05 [92.12, 108.77], *p* = 0.017. HMF-AL, human milk fortifier-acidified liquid; IQR, interquartile range; LHMF-NEW, new, liquid human milk fortifier.

The proportion of infants receiving fortification decreased progressively over time and was not different between groups. About 93 to 96% of infants received fortified human milk 2 weeks after starting fortification; this proportion decreased to between 78 and 88% at 3 weeks and between 55 and 68% at 4 weeks. Thereafter, fewer infants in either group received fortified human milk (one center stopped using DHM after 30 days). Total fluid intake did not differ between groups at the start or during the several weeks of fortification ( Fig. 1A ). The median protein intake before fortification started was approximately 2.7 g/kg/d in both groups and subsequently increased with small but significantly higher total protein intakes in the HMF-AL group at 1 ( *p* <0.001), 2 ( *p* = 0.015), and 3 ( *p* = 0.006) weeks of fortification ( Fig. 1B ). A few infants had markedly high protein intakes primarily due to large intake volumes, especially when receiving fortification for more than 2 weeks. A marginal but significant difference was detected in overall caloric intake between groups before fortification started ( *p* = 0.017, Fig. 1C ). Feeding intolerance was uncommon and occurred in equal proportion within each week of fortification using either fortifier (week 1 HMF-AL 3%, LHMF-NEW 4%; week 2 HMF-AL 8%, LHMF-NEW 6%; week 3 HMF-AL 5%, LHMF-AL 5%, and week 4 HMF-AL 2%, LHMF-NEW 3%).

### Growth Outcomes

**Fig. 2:**
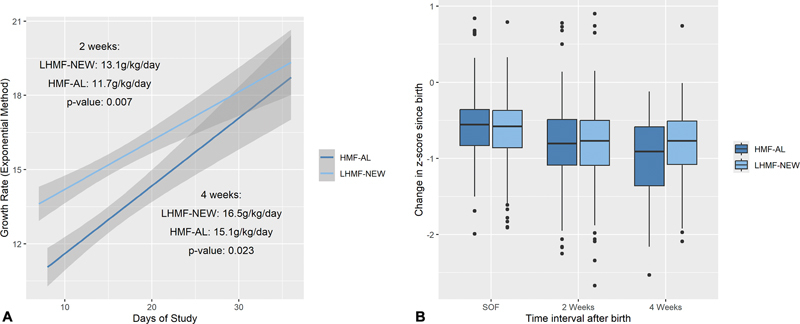
( **A** ) Growth rate over the study period calculated with the exponential method. Comparisons of the growth velocity regression lines for the HMF-AL (blue) and LHMF-NEW (light blue) groups were done using a repeated measures regression (see Methods) only including infants who received either fortifier throughout the first 4 weeks of treatment ( *N* = 308). Gestational age, fortifier group, location, and gender were incorporated into the model. ( **B** ) Changes in weight Z-score over time for the groups receiving HMF-AL (blue) or LHMF-NEW (light blue) are shown as median and 25 to 75 IQR; vertical lines represent the range. No significant differences were identified. HMF-AL, human milk fortifier-acidified liquid; IQR, interquartile range; LHMF-NEW, new, liquid human milk fortifier; SOF, start of fortification.

Growth velocity in weight at 2, 3, and 4 weeks of fortification was significantly higher in the group receiving LHMF-NEW ( Fig. 2A ). This group also exhibited a higher weight growth velocity at 5 weeks of fortification, but the overall proportion of infants receiving fortified human milk had dropped markedly. When weight growth velocity was compared among infants that received either fortifier for all 4 weeks ( *N* = 308) using a regression model including gestational age, fortifier group, location, sex, and use of supplemental oxygen or respiratory support, the effect of LHMF-NEW remained significant (2.10, confidence interval: 0.83–3.36 g/kg/d, *p* = 0.001). Z scores for weight, length, and head circumference decreased comparably over time in both groups, although at 4 weeks of fortification, the decrease in weight Z scores was less pronounced in the LHMF-NEW group ( Fig. 2B ). There was no significant difference in overall weight growth rate among infants with or without metabolic acidosis (15.6 vs. 16.4 g/kg/d, respectively, *p* = 0.15). Discharge medians [25–75% IQ] for weight, length, and head circumference were comparable between groups (2,670 [2,286, 3,173] vs. 2,635 [2,302, 3,170] g; 45.6 [43.5, 47.5] vs. 45.1 [43.5, 47.0] cm; 33.0 [31.5, 34.5] cm vs. 33.0 [31.5, 34.5] cm, for the HMF-AL and LHMF-NEW groups, respectively).

## Discussion

Fortification of human milk is critical to ensure that VLBW infants receive an appropriate intake of protein and other nutrients needed for optimal growth. 3 4 5 Currently, there are several commercially available fortifiers of human or bovine milk origin in liquid or powder form, which vary in their nutrient composition, although the HMF-AL is no longer in clinical use. 8 At present, the evidence does not support better outcomes with human milk-derived fortifiers over bovine products. 9 10 This notwithstanding, it is very important to examine clinical outcomes, particularly growth, among populations of infants receiving these fortifiers. In a blind, randomized trial, Moya et al showed that using the HMF-AL resulted in better growth compared with a powdered form of fortification with a lower protein content. 11 Also, in an unblinded, randomized trial, Kim et al reported better growth with a liquid formulation of a bovine milk-derived fortifier with extensively hydrolyzed protein compared with a powdered form with less protein. 12 Both these bovine milk-derived liquid fortifiers were compared in an unblinded, randomized trial by Schanler et al, which showed similar growth velocities during a 4-week fortification period. 14 These growth velocities were comparable to those we identified in our study. Several randomized trials using human milk-derived fortifiers have been conducted, which seem to show a slower growth velocity when compared with that observed with bovine-derived fortifiers. 6 Only with the addition of human milk cream as an energy supplement to standard fortification have better growth rates been achieved. 18

In this study, we report growth outcomes in a large population of VLBW infants receiving a new bovine-derived fortifier compared with infants receiving a previous formulation of this fortifier already studied in clinical trials, but no longer available for use. 11 We examined these outcomes in three NICUs from separate regions, managed by different providers using similar entry criteria to previous controlled trials of bovine-derived fortifiers. 11 12 14 This “real-world” approach could enhance the generalizability of our findings. There were significant albeit generally small differences in many clinical characteristics between infants from the participating centers, including the median days of age when fortification was initiated where one center added fortification many days before the other two NICUs ( Table 2 ). This reflects each NICU's feeding guidelines and the lack of a widely accepted approach to nutritional support in VLBW infants ( Supplementary Material S1 , available in the online version). However, when infants from all three NICUs were grouped according to which fortifier they received, the groups were comparable, except for whether participants were receiving supplemental oxygen or noninvasive respiratory support ( Table 3 ). This variable as well as sex were incorporated into regression models to calculate growth velocity.

The majority of VLBW infants received fortified MOM or DHM for several weeks and the proportion of infants receiving DHM was similar between groups when all three NICUs were compared separately or once grouped according to the fortifier received. 2 7 14 19 Likewise, in the NICU that utilized HMBP to initiate feedings, the proportion of infants in the HMF-AL and LHMF-NEW groups that received this was similar. Moreover, the proportion of infants that received fortification for 4 weeks is similar to what has been reported previously in clinical trials of bovine-derived fortifiers. 11 12 14

Growth velocity was higher among infants receiving the new LHMF-NEW during the first several weeks of fortification. This benefit persisted beyond 4 weeks of fortification; however, there was considerable attrition in the number of infants still receiving either fortifier past this time. In addition, when growth velocity was compared among only those infants that received either fortifier for 4 weeks, a common outcome reported in several previous fortifier studies, 11 12 14 significantly greater weight growth velocity was maintained in infants receiving LHMF-NEW. This was observed despite the fact that more infants in the LHMF-NEW group were still on oxygen or receiving respiratory support at 4 weeks of fortification. This is relevant since VLBW infants with developing or established chronic lung problems tend to grow slower. 20 Z-scores also suggested an advantage in weight gain for the LHMF-NEW group. Typically, weight Z scores decrease across the length of an NICU hospitalization in VLBW infants; however, the decrease in weight Z scores was less in infants receiving LHMF-NEW. Higher weight gain for the LHMF-NEW group was observed, despite similar intakes of total fluids and calories between both groups, and a slightly higher protein intake among infants fed the HMF-AL. We focused mainly on weight gain because measurements of length can be variable if not using length boards. This was the case in one of our NICUs. Growth velocity in both groups, but particularly in the LHMF-NEW group is comparable to that reported in recent controlled trials of bovine-based fortifiers and appears to be higher than when human milk-derived fortifiers are used. 11 12 14 18 19 Achieving a better growth velocity during their NICU stay is of paramount importance for VLBW infants, given the association of slower growth velocity with a higher risk for abnormal neurodevelopmental outcome as reported by Ehrenkranz et al. 21

Why growth velocity was higher among infants receiving the LHMF-NEW is unclear since the volume or energy intake did not differ between groups during fortification. There was a slightly higher protein intake in the group receiving the HMF-AL, whereby excess protein in the setting of renal immaturity may have led to more metabolic acidosis and perhaps slower growth. 22 Feeding intolerance was also not a factor, since it was reported with similar frequency in either group. Additional morbidity is unlikely to explain the difference in growth velocity since complications like sepsis and NEC did not differ between fortifier groups. Furthermore, more infants in the LHMF-NEW group were in oxygen or respiratory support, yet they grew faster.

Metabolic acidosis is common among preterm infants and relates, in part, to protein intake and renal immaturity. 22 23 Neonatologists often add early in the NICU course acetate salts to parenteral nutrition or umbilical arterial solutions to buffer any decrease in pH and base deficit observed in preterm infants. Metabolic acidosis has been associated with slower growth and a longer length of stay, although this did not occur in our study. 14 24 25 26 27 28 29

There was a trend toward a shorter length of stay and earlier corrected gestational age at discharge, although these differences were not significant. This is not surprising given that the decision to discharge an infant depends on many factors and not only on their nutritional/growth status. Furthermore, the lack of differences in anthropometrics at discharge between groups suggests that any growth advantage observed during fortification may no longer be observed once this stops or is modified. 11 12 30 31 There is enormous variability in nutritional practices after a period of fortification, and human milk fortifiers may not be added after certain postnatal age or corrected gestational age is achieved, or in preparation for discharge. No differences between the HMF-AL and LHMF-NEW groups were observed in late-onset sepsis or NEC. Both these complications occurred with relatively low frequency as recently reported in large databases, 32 and as one might expect in a stable population of VLBW infants receiving human milk as their main source of nutrition. 7 11 12 14 19 33

Even though we sought to compare these fortifiers in a “real-world” setting to improve the generalizability of our study, this approach also has several limitations. Given our study's retrospective design spanning several years, practice modifications such as more aggressive enteral nutritional support may have occurred during this time period that would be hard to ascertain retrospectively. 30 Also, important differences were noted between centers in the timing of starting trophic feedings and fortification, in addition to the use of HMBP in one center. These differences may have impacted some of our findings. However, we attempted to address this by including some of these factors in our statistical analysis.

## Conclusion

In the current study, in a large population of VLBW infants receiving a new commercial bovine-based liquid human milk fortifier (vs. the previous human milk fortifier-acidified liquid), growth velocity was significantly higher at 2, 3, and 4 weeks of receiving fortification, continued to be higher through week 5 in infants still receiving fortified milk, and was significantly higher in infants that received fortified milk for at least 4 weeks. The incidence of feeding intolerance (stopping feeding > 8 hours) was low in both groups. Furthermore, the incidence of late-onset sepsis was low, no differences in NEC were detected between groups, and the incidence of metabolic acidosis was lower in infants receiving the LHMF-NEW. No group differences in length of NICU stay or anthropometrics at discharge were detected. Evaluation and reporting of clinical experiences of human milk fortifier use, especially in VLBW infants, is important to continue understanding how best to support nutrition and growth of preterm infants.
