# Supplementary material for: Improved Growth Velocity Using a New Liquid Human Milk Fortifier in Very Low Birth Weight Infants: A Multicenter, Retrospective Study
Source: Am J Perinatol. 2025 Mar 6;42(13):1729–37. doi: 10.1055/a-2527-4638 (PMC12431800; doi:10.1055/a-2527-4638)
Supplement: Supplementary file 1 — Supplementary Material [file 10-1055-a-2527-4638-s24jul0431.pdf]

Supplementary Material S1 Feeding guidelines by institution

ECU Health

<1,500 g (aim to start feeds by DOL #1)

Feeding day	mL/kg/d	Feed frequency	High-protein HMF	IV lipids	Notes
1	20	Every 3 h		1 g/kg	Donor or MBM
2	20	Every 3 h		2 g/kg	
3	20	Every 3 h		3 g/kg	
4	40	Every 3 h		3 g/kg	
5	60	Every 3 h		2 g/kg	
6	80	Every 3 h	22 kcal	1 g/kg	
7	100	Every 3 h	24 kcal	0 g/kg	
8	120	Every 3 h	24 kcal		d/c TPN
9	140	Every 3 h	24 kcal		
10	150–160	Every 3 h	24 kcal		Start vitamin D

Palm Beach Children's Hospital

<1500 g (aim to start feeds by DOL #3)

Feeding day	mL/kg/d	Feed frequency	Fortification	Other
1–3	10–20	Every 3 h		MBM or prolacta donor milk
3	10–20	Every 3 h		Advance if tolerating
4	30–40	Every 3 h		
5	50–60	Every 3 h		
6	70–80	Every 3 h	+4 kcal/oz	Prolacta
7	90–100	Every 3 h	+4 kcal/oz	D/C lipids
8	110–120	Every 3 h	+4 kcal/oz	D/C TPN
9	130–140	Every 3 h	+4 kcal/oz	
10	150–160	Every 3 h	+4 kcal/oz	
11	150–170	Every 3 h	+4 kcal/oz	Increase to +6 or +8 if needed
32 wk or >1,250 g	150–170	Every 3 h	MBM w/ HMF SP or formula	D/C prolacta<br>Over 4-d transition

*If mother's own breast milk is not available then we use Prolacta's ready to feed (RTF) until 32 weeks.

 © 2025. The Author(s).

Betty Cameron Children's Hospital

<750 g (Aim to start feeds by DOL #4)

Day of feeding	mL/kg/d	Feed frequency	Notes
1	10	Every 3 h	×1 day, donor or MBM
2–6	20	Every 3 h	×5 day
7	40	Every 3 h	
8	60	Every 3 h	
9	80	Every 3 h	Consider decreasing lipids by 50%
10	100	Every 3 h	D/C lipids, fortify EBM (22 kcal/oz)
11	120	Every 3 h	
12	140	Every 3 h	D/C TPN, fortify EBM (24 kcal/oz)
13	160–170	Every 3 h	

750–1,000 g (aim to start feeds by DOL #4)

Day of feeding	mL/kg/d	Feed frequency	Notes
1–4	20	Every 3 h	×4 d, donor or MBM
5	40	Every 3 h	
6	60	Every 3 h	
7	80	Every 3 h	Consider decreasing lipids by 50%
8	100	Every 3 h	D/C Lipids, fortify EBM (22 kcal/oz)
9	120	Every 3 h	
10	140	Every 3 h	D/C TPN, fortify EBM (24 kcal/oz)
11	160–170	Every 3 h	

1,001–1,500 g (aim to start feeds by DOL #4)

Day of feeding	mL/kg/d	Feed frequency	Notes
1–3	20	Every 3 h	×3 d, donor or MBM
4	40	Every 3 h	
5	60	Every 3 h	
6	80	Every 3 h	Consider decreasing lipids by 50%
7	100	Every 3 h	D/C lipids, fortify EBM (23 kcal/oz)
8	120	Every 3 h	
9	140	Every 3 h	D/C TPN, fortify EBM (24 kcal/oz)
10	160–170	Every 3 h	

Abbreviations: D/C, discharge; DOL, day of living; EBM, expressed breast milk; HMF, human milk fortifier; IV, intravenous; MBM, maternal breast milk; SP, standard protein; TPN, total parenteral nutrition.

 © 2025. The Author(s).
